# Secondary care intervals before and after the introduction of urgent referral guidelines for suspected cancer in Denmark: a comparative before-after study

**DOI:** 10.1186/1472-6963-13-348

**Published:** 2013-09-10

**Authors:** Mette Bach Larsen, Rikke Pilegaard Hansen, Dorte Gilså Hansen, Frede Olesen, Peter Vedsted

**Affiliations:** 1 Research Unit for General Practice, Aarhus University, Bartholins Allé 2, DK-8000 Aarhus C, Denmark; 2 Section for General Practice, Department of Public Health, Aarhus University, Bartholins Allé 2, DK-8000 Aarhus C, Denmark; 3 Centre for Cancer Diagnosis in Primary Care – CaP, Aarhus University, Bartholins Allé 2, DK-8000 Aarhus C, Denmark; 4 National Research Centre for Cancer Rehabilitation, Research Unit for General Practice, University of Southern Denmark, J.B. Winsløws vej 9, DK-5000 Odense C, Denmark

**Keywords:** Denmark, Early detection of cancer, Family practice, Health services administration

## Abstract

**Background:**

Urgent referral for suspected cancer was implemented in Denmark on 1 April 2008 to reduce the secondary care interval (i.e. the time interval from the general practitioner’s first referral of a patient to secondary health care until treatment is initiated). However, knowledge about the association between the secondary care interval and urgent referral remains scarce. The aim of this study was to analyse how the secondary care interval changed after the introduction of urgent referral.

**Methods:**

This was a retrospective population-based study of 6,518 incident cancer patients based on questionnaire data from the patients’ GPs. Analyses were stratified with patients discharged from Vejle Hospital in one stratum and patients from other hospitals in another because Vejle Hospital initiated urgent referrals several years prior to the national implementation. Further, analyses were stratified according to symptom presentation and whether or not the GP referred the patient on suspicion of cancer. Symptom presentation was defined as with or without alarm symptoms based on GP interpretation of early symptoms.

**Results:**

The median secondary care interval decreased after the introduction of urgent referral. Patients discharged from Vejle Hospital tended to have shorter secondary care intervals than patients discharged from other hospitals. The strongest effect was seen in patients with alarm symptoms and those who were referred by their GP on suspicion of cancer. Breast cancer patients from Vejle Hospital experienced an even shorter secondary care interval after the national introduction of urgent referrals.

**Conclusion:**

Urgent referral had a positive effect on the secondary care interval, and Vejle Hospital remarkably managed to shorten the intervals even further. This finding indicates that the shorter secondary care intervals not only result from the urgent referral guidelines, but also involve other factors.

## Background

The lower cancer survival rates in Denmark than in the other Nordic countries and many European countries have been the focus of much attention since the beginning of this century [1-4]. Studies have indicated that part of the explanation is that Danish cancer patients seem to be at a more advanced stage of disease than patients in the other Nordic countries when treatment is initiated [5,6]. Their diagnosis and treatment may be delayed due to prolonged diagnostic and treatment intervals, defined as the period of time from first cancer symptom until diagnosis and treatment [7]. The consequences of prolonged diagnostic and treatment intervals have long been a controversial issue [8,9], but a recent study suggested a plausible explanation for disparities among previous results and showed that a longer diagnostic interval was associated with increased mortality among colorectal cancer patients [10].

The time interval from the first referral from primary care to treatment may be referred to as the secondary care interval [7]. A long secondary care interval was documented in Denmark in 2007 [11-13], which directed political attention to the waiting lists in the Danish health care sector and made the Danish government and the Danish regions (who own and run the hospitals) launch a new diagnostic strategy that classified cancer as an acute condition. This marked the beginning of a re-organisation of the cancer care pathway in Denmark [14].

The Danish government launched its second cancer plan in 2005, which prescribed the use of a standardised pathway from cancer suspicion until treatment [15]. In the summer of 2007, the government decided to implement at a national level the urgent referral pathway which had been successfully pursued at regional level at Vejle Hospital since the late 90s. The principle of urgent referral was formally introduced for breast, lung, colorectal and head and neck cancers on a nationwide basis on 1 April 2008. Gynaecological cancers followed on 1 August 2008 and haematological cancers on 1 September 2008. By 2010, the urgent referral scheme had been introduced for 34 different cancers [16]. Urgent referral consists of a set of standardised procedures aiming at offering patients the optimal diagnostic process and rapid treatment [15].

In Denmark, general practitioners (GPs) serve as gatekeepers to secondary health care. For urgent referrals to work, the GPs must suspect a specific type of cancer on the basis of a predetermined set of alarm symptoms. However, one study has shown that only approx. 50% of all cancer patients present with typical alarm symptoms in general practice [17]. This may be explained by the fact that alarm symptoms of cancer are difficult to define and that symptoms traditionally considered as alarm symptoms (e.g. rectal bleeding, lumps or haemoptysis) often have positive predictive values of less than 5% [18]. International experience has even indicated that urgent referrals may have adverse effects on waiting times for patients without alarm symptoms [19,20].

Thus, urgent referral was introduced nationally under the assumption that the secondary care interval would be reduced. However, knowledge about the actual association between the secondary care interval and urgent referrals is scarce. Therefore, the aim of this study was to analyse how the secondary care interval changed, in general and at Vejle Hospital in particular, after the introduction of urgent referral for specific types of cancer.

## Method

### Study design and setting

This population-based observational study was conducted among incident cancer patients in the Central Denmark Region and the Region of Southern Denmark. The two regions in combination have approx. 2.4 million residents (44% of the Danish population) and approx. 14,000 new cancer cases are diagnosed each year.

Vejle Hospital is situated in the Region of Southern Denmark and the hospital’s oncology department constitutes one of six Danish cancer centres. The oncology department performs 38,000 outpatient consultations per year, whereas inpatient bed days amount to 7,500 per year.

Denmark’s publicly funded healthcare system provides free access to general practice and hospital care. More than 98% of the Danish citizens are registered with a GP. Danish GPs keep electronic medical records of their patients, including hospital discharge letters.

### Inclusion procedure

**Figure 1:**
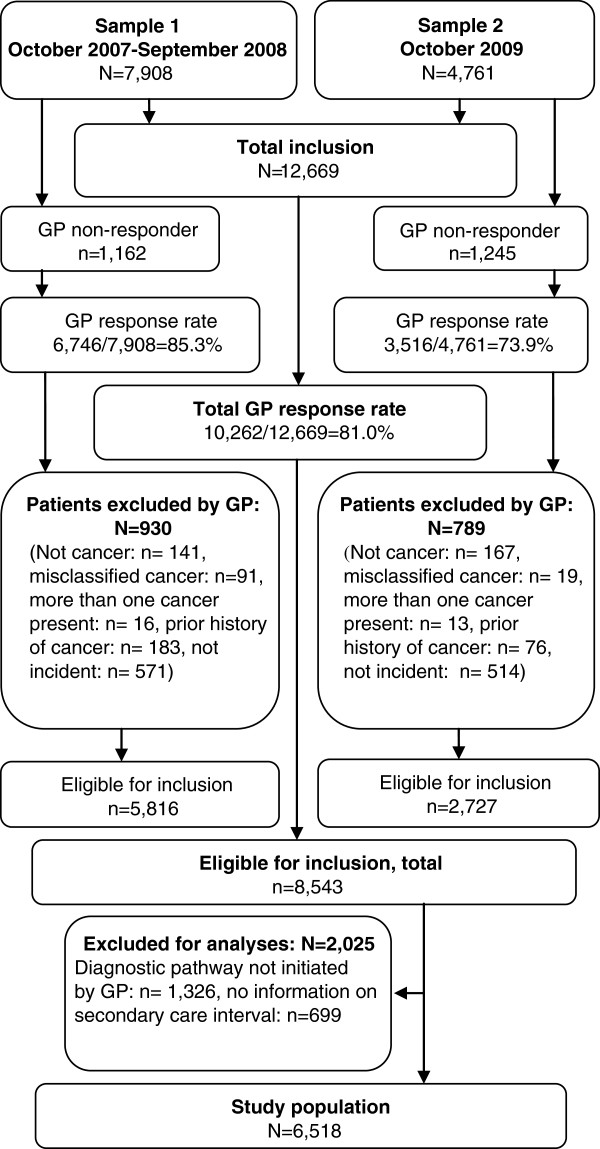
Flowchart of patient inclusion.

Each hospital admission and outpatient visit in Denmark is coded and stored in regional patient-administrative systems, which feed the National Patient Register [21]. Patients were included from the patient administrative systems on the basis of their discharge date and diagnosis and if labelled with the additional code AZCA1, which indicates that the cancer was reported for the first time by the department [22]. Both outpatients and patients dying under admission are registered with a discharge date. Patients were excluded if already registered on a national list including all cancer diagnoses from 1994 to 30 September 2007, which was extracted from the National Patient Registry. This list was updated monthly by adding included patients. The monthly inclusion continued for one year from 1 October 2007 and constitutes Sample 1 of the study population. After the study period ended, it became clear that some of the eligible patients were excluded for two main reasons. First, some of the patients were registered later than one month after the diagnosis and were missed as the inclusion procedure only included one month back. Second, the AZCA1 code was applied in an unsystematic way causing inclusion errors. Therefore, additional inclusion was performed in October 2009 by following the same procedure as Sample 1, except that the entire study period was covered at once and the AZCA1 code was left out as an inclusion criterion (Sample 2). Patients were excluded from Sample 2 if already included in Sample 1. Thus, the total study population consists of Sample 1 and Sample 2 (Figure 1).

### Data collection

The GP questionnaire was developed based on literature and research experience gained from prior studies of patients’ diagnostic pathways [23]. For patients from Sample 1, the GPs were sent a questionnaire within a month after the patients were diagnosed. For patients in Sample 2, the GPs received the questionnaires in October 2009. The GPs provided information on the date of their first referral of the patient to secondary health care and the date of treatment start based on their medical records and discharge letters.

The GPs also stated whether the diagnostic pathway was initiated in general practice, whether they regarded the initial symptoms as alarm symptoms of cancer and whether they clearly indicated cancer suspicion in the first referral to secondary health care. Alarm symptoms of cancer were not defined in the questionnaire, but the GPs were guided by examples of unexpected weight loss, bleeding, continuous coughing and lumps.

Non-responding GPs received a reminder after three weeks. The GPs were remunerated for their participation (approx. 16 €).

### Outcome measures

Estimation of the secondary care interval was primarily based on data obtained from the GP questionnaires. The GPs provided information on when they initially referred the patients to secondary health care and stated the date of treatment based on their medical records.

However, for 2,024 (31.1%) patients, the GPs did not provide complete information on the secondary care interval, mainly because the GPs had received the questionnaire before obtaining information about treatment start. For those patients, we estimated the secondary care interval on the basis of the patients’ hospital admission date. This approximation systematically underestimated the interval as the median GP-reported secondary care interval was 40 days (IQI: 22; 74 days) compared with the 31-day (IQI: 14; 66 days) interval obtained when the interval was calculated on the basis of the patients’ date of admission.

Specific analyses were performed for patients diagnosed before or after the implementation of urgent referral for suspected cancer if they were admitted to hospital before or after 1 April 2008, respectively. Patients were further divided into groups according to whether the GPs interpreted the initial symptoms as alarm symptoms of cancer and whether cancer suspicion was clearly mentioned in the first referral. Finally, patients were grouped according to discharging hospital with Vejle Hospital in one group and other hospitals in another.

### Analyses

Data analyses were restricted to include only patients with a diagnostic pathway initiated in primary care since the pathway of interest in the present study was the part where GPs refer patients to further diagnostic work-up in secondary care (main route to diagnosis of cancer in Denmark [23]).

The secondary care interval is presented as medians with interquartile intervals (IQIs) because data were not normally distributed. The Wilcoxon rank sum test was used to test for differences before and after the national introduction of urgent referral for suspected cancer. Trends in monthly median differences were identified by non-parametric trend tests across ordered groups. To test for differences between groups, the Wilcoxon rank sum test was used. Estimates were given with 95% confidence intervals (95%CI) when relevant. Analyses were conducted using Stata 11.2 [24].

### Ethics approval

According to the Scientific Ethics Committee in the former County of Aarhus, the project did not need approval by the Danish Biomedical Research Ethics Committee System. The study was approved by the Danish Data Protection Agency and the Danish National Board of Health.

## Results

A total of 12,669 cancer patients were screened for inclusion into the study (Sample 1: n = 7,908; Sample 2: n = 4,761), and a GP questionnaire was completed for 10,262 (81.0%). Based on the questionnaires, a total of 1,719 (16.8%) patients were excluded for not meeting the inclusion criteria. Of the remaining 8,543 patients eligible for inclusion, the diagnostic pathway was not initiated through the GP for 1,326 of the (15.5%) patients. They were excluded together with 699 (8.2%) patients for whom we had no information on the secondary care interval. Thus, a total of 6,518 (59.5%) patients were included in the analyses (Figure 1).

Patients with non-responding GPs were more likely to be older males, fewer had breast and lung cancers and more had prostate cancer. No statistically significant difference was observed in response rate from GPs with patients discharged from Vejle Hospital compared with other hospitals. Patients discharged from Vejle Hospital were statistically significantly more likely to be females, younger and diagnosed with breast cancer. Secondary care intervals were statistically significantly more often incomplete in other hospitals than Vejle Hospital, in patients without alarm symptoms and in cases when the GPs did not indicate cancer suspicion in the referral papers (Table 1). No differences were observed in the proportion of incomplete secondary care intervals before and after the introduction of urgent referral for suspected cancer (data not shown).

**Table 1:** Patient characteristics of those discharged from Vejle hospital and other hospitals, respectively

	**Other hospitals n (%)**	**Vejle hospital n (%)**	
**All**	5,743 (88.1)	775 (11.9)	p-value
**Sex**			
Male	2,917 (50.8)	268 (34.6)	<0.001
Female	2,826 (49.2)	507 (65.4)	
**Age (years)**			
18-49	690 (12.0)	124 (16.0)	<0.001
50-69	2,656 (46.3)	294 (51.4)	
70+	2,397 (41.7)	253 (32.7)	
**Cancer diagnosis**			
Breast	772 (13.4)	260 (33.6)	<0.001
Colorectal	842 (14.7)	96 (12.4)	0.090
Lung	712 (12.4)	113 (14.6)	0.086
Head and Neck	165 (2.4)	9 (1.2)	0.006
Prostate	746 (13.0)	19 (2.5)	<0.001
Other	2,506 (43.6)	278 (35.9)	<0.001
**Alarm symptoms**			
Yes	3,541 (62.7)	519 (67.8)	0.007
No	2,104 (37.3)	247 (32.3)	
**Cancer suspicion**			
Yes	3,156 (63.6)	435 (66.4)	0.154
No	1,809 (36.4)	220 (33.6)	

### The secondary care interval before and after the introduction of urgent referral for suspected cancer

The secondary care interval was statistically significantly shorter at other hospitals after the national introduction of urgent referral for suspected cancer than before the introduction. Breast cancer patients saw the largest reduction (10 days), whereas no statistically significant reduction was found for head and neck cancer patients. At Vejle Hospital, the improvement was statistically significant only for breast cancer (eight days) (Table 2).

**Table 2:** The median secondary care interval (days) before and after urgent referral for suspected cancer

	**Other hospitals**			**Vejle hospital**		
	**Before urgent referral n = 3,131**	**After urgent referral n = 2,612**		**Before urgent referral n = 387**	**After urgent referral n = 388**	
	**Median (IQI)**	**Median (IQI)**	**p-value**	**Median (IQI)**	**Median (IQI)**	**p-value**
**All**	42 (22; 80)	35 (18; 67)	<0.001	30 (20; 50)	26 (15; 51)	0.020
**Sex**						
Male	46 (23; 90)	40 (20; 80)	<0.001	32 (20; 63)	29 (17; 68)	0.362
Female	39 (21; 69)	32 (16; 57)	<0.001	29 (20; 45)	26 (15; 48)	0.036
**Age (years)**						
18-49	41 (21; 73)	29 (15; 61)	0.003	35 (18; 61)	25 (14; 39)	0.132
50-69	53 (30; 98)	45 (22; 81)	<0.001	29 (20; 43)	26 (17; 50)	0.408
70+	41 (21; 77)	36 (18; 68)	<0.001	33 (22; 56)	27 (15; 52)	0.062
**Cancer diagnosis**						
Breast	33 (21; 56)	23 (15; 38)	<0.001	28 (20; 40)	21 (13; 33)	<0.001
Lung	37 (21;64)	33 (16; 53)	0.008	31 (20; 41)	29 (23; 65)	0.392
Colorectal	38 (22; 71)	30 (18; 53)	<0.001	32 (23; 46)	26 (18; 37)	0.068
Head and neck*	39 (22; 64)	37 (17; 66)	0.443			
Prostate*	72 (37; 141)	67 (31; 104)	0.028			
Other	42 (19; 80)	40 (18; 76)	0.358	30 (17; 64)	31 (16; 70)	0.842
**Alarm symptoms**						
Yes	38 (21; 70)	33 (16; 58)	<0.001	32 (22; 49)	27 (18; 44)	0.025
No	49 (26; 96)	42 (20; 86)	<0.001	41 (27; 89)	36 (18; 77)	0.097
**Cancer suspicion**						
Yes	36 (20; 67)	30 (16; 56)	<0.001	30 (21; 44)	22 (15; 37)	0.001
No	52 (27; 98)	46 (23; 89)	0.018	51 (30; 91)	43 (28; 84)	0.259

*The number of cases at Vejle Hospital was too small to produce precise results on head and neck cancer (n = 9) and prostate cancer (n = 19).

At other hospitals than Vejle, the median secondary care interval decreased regardless of whether the GP indicated cancer suspicion or alarm symptoms of cancer. At Vejle Hospital this was only the case if the GPs indicated cancer suspicion or alarm symptoms in their referral documents (Table 2).

For diagnoses where the urgent referral policy was introduced after the study period, the secondary care interval did not decrease significantly (other diagnosis, Table 2).

### Trends in secondary care interval by month

**Figure 2:**
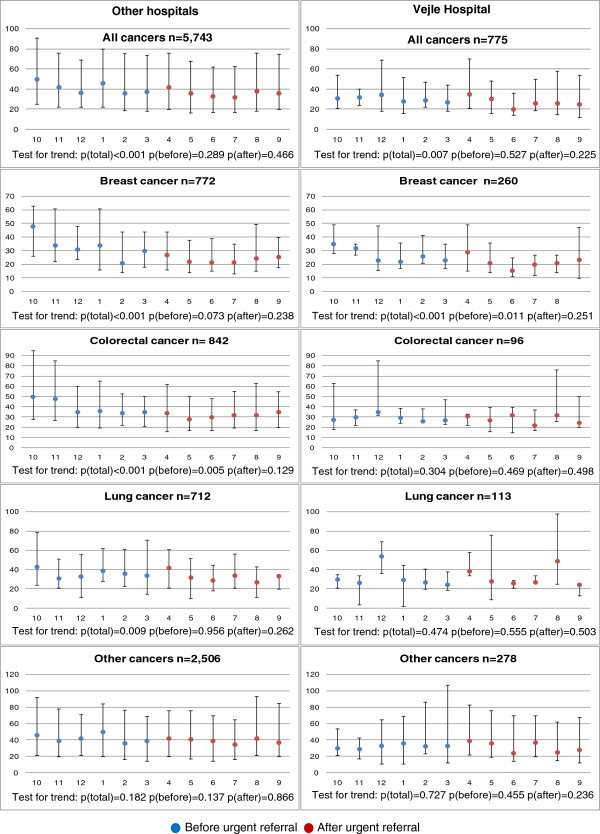
**Monthly secondary care intervals (median days with interquartile intervals).** Urgent referral for suspected cancer was introduced in April 2008.

The median secondary care interval for the study period for all patients discharged from Vejle Hospital was 29 days (IQI: 18; 50 days) compared with 39 days (IQI: 20; 74 days) for patients discharged from other hospitals. For both Vejle Hospital and other hospitals, a statistically significantly decreasing trend was observed (Vejle: p = 0.007, other: p < 0.001). Stratified by diagnosis, the decreasing trend in the median secondary care interval was statistically significant for breast cancer (p < 0.001), colorectal cancer (p < 0.001) and lung cancer (p = 0.009) at other hospitals, but only for breast cancer at Vejle Hospital (p < 0.001). No statistically significant decreasing trend was observed for other cancer diagnoses. Further, a tendency towards a decrease in the secondary care interval was seen for other hospitals than Vejle Hospital before the introduction of urgent referral. This tendency was statistically significant for colorectal cancer (Figure 2).

## Discussion

### Main findings

The median secondary care interval decreased overall after the introduction of urgent referrals for suspected cancer. With a decrease of 10 days, breast cancer patients saw the largest decrease. Studies have shown that breast cancer patients with treatment delays of three months or more had a lower 5-year survival rate than patients with shorter delays [25]. Our finding of a median reduction of 10 days may not result in reduced mortality in general, but the patients who waited the longest (e.g. the 75 percentile was 74 days at other hospitals than Vejle before the national introduction of the urgent referral guidelines) may have gained more from the introduction of the urgent referral guidelines. Finally, there may be other benefits valued by patients as well as professionals.

Both before and after the national introduction of the urgent referral guidelines, patients had a shorter secondary care interval in cases where initial symptoms were categorised as alarm symptoms by the GP or the GP indicated cancer suspicion in the referral documents. Obviously, patients with a clearer pathological picture are easier to diagnose. This also supports the assumption that the GP’s symptom interpretation and reaction to suspicion of cancer is instrumental in shortening the secondary care interval for cancer patients. However, at other hospitals than Vejle, the secondary care interval also decreased for patients with no indication of cancer suspicion and no alarm symptoms, indicating improved performance of the system as a whole.

The tendency towards a decrease in the secondary care interval at other hospitals seemed to appear when the need for such decrease was officially recognised, i.e. before the formal implementation of the urgent referral guidelines. Furthermore, it should be noted that Vejle Hospital was able to reduce its secondary care intervals even further when the introduction of the urgent referral system was introduced at national level.

The study design does not allow any determination of causation. The fact that diagnoses without urgent referrals did not decrease statistically significant after the introduction of urgent referrals indicates that the decreasing secondary care intervals could be related to the introduction of urgent referrals. On the contrary, the fact that Vejle Hospital actually managed to decrease their secondary care intervals, also for patients with no indication of cancer suspicion in the referrals from their GP, indicates that the decrease is also related to other factors.

### Strengths and weaknesses

Selection bias in this study was induced during the initial inclusion procedure. We handled this by adding a second inclusion and hereby completing the cohort of patients. However, this may have induced information bias since the GPs of Sample 1 received the questionnaire within one month after diagnosis, while the GPs of Sample 2 received the questionnaire between one and two years after diagnosis. GP recall bias could influence the secondary care interval and the categorisation of patients according to initial symptom presentation and cancer suspicion for the first referral to secondary health care. Recall bias is not considered to have much impact on length of secondary care interval nor on number of suspected cancer referrals since the GPs have access to this information through the patients’ electronic medical records, which are considered robust data sources. Apart from guiding the GPs with examples of alarm symptoms, it was not specified precisely which symptoms should be categorised as alarm symptoms. Thus, the interpretation of the same symptom could differ among GPs. This source of information bias is not considered to be major for two reasons. First, alarm symptoms of cancer are not clear-cut and will therefore be influenced by individual GP interpretation. Second, alarm symptoms of cancer are known to the GPs so that if they do suspect cancer such symptoms (e.g. rectal bleeding) will be categorised as alarm symptoms. Another possible source of information bias is the retrospective design of the study. The GP knows that the patient has cancer when filling out the questionnaire which may influence the answers. However, it would be costly to set up a prospective study given the few cancer patients diagnosed by each GP every year. Furthermore, it has been shown that the GPs are willing to answer these questions because they see a learning potential in going through the care pathway retrospectively [26]. In conclusion, even though the retrospective design may be a source of bias, the data are considered a reliable foundation for the analyses.

GP-induced selection bias may have occurred if patients of non-responding GPs had a different secondary care interval than patients of responding GPs. If GPs with a longer doctor interval (time from the patient’s first contact with the GP until first cancer-related investigation) tended to be more reluctant to respond, this could lead to shorter secondary care intervals. This bias may therefore lead us to make a type II error by underestimating the association between the introduction of the urgent referral guidelines and the secondary care interval.

The considerable sample size strengthens the statistical precision of this study. We did not have complete information on the secondary care interval for almost one third of the patients due to missing treatment dates. We used the date of admission in these cases, which introduced a systematic underestimation of the secondary care interval. Since more patients in other hospitals than Vejle as well as patients without alarm symptoms and without indication of cancer suspicion had incomplete secondary care intervals, we underestimated the secondary care interval for patients with the longest secondary care interval, and this may have provided absolute minimum differences. Since we were still able to detect considerable changes, we have found that this bias was not considerable enough to significantly alter our conclusions.

No information is available on who were actually referred urgently after the introduction of urgent referral. The GPs’ reported data on whether patients presented with alarm symptoms and clearly indicated cancer suspicion in connection with first referral are therefore our best sources of information for determining whether the patients were regarded as urgent or not. However, since the study included a period before and a period after the urgent referral was implemented, it is meaningful to compare the GPs’ assessment of the initial symptoms and whether or not the GPs indicated cancer suspicion in their referrals to secondary health care since we have this information for both periods.

The conclusions of this study are considered generalisable to other regions in Denmark and to other health care systems involving GPs serving as gatekeepers to secondary health care. The possible impact of the urgent referral will depend on the local context.

### Findings in relation to other studies

The overall median secondary care interval of 42 days (IQI: 22; 80 days) for other hospitals than Vejle Hospital before the introduction of urgent referrals is fairly consistent with the results from another Danish study from 2005, which reported a secondary care interval of 46 days (IQI: 26;78) [13]. This study used the same definition of the secondary care interval and also collected data by use of questionnaires sent to the GPs. A comparison of our results with the findings of a recent study by the Danish National Board of Health shows that the secondary care intervals reported in our study were shorter than those previously reported, except for colorectal cancer [12]. The National Board of Health counted the days from the receipt of referrals at the hospitals, whereas we measured from the day on which the referrals were forwarded by the GPs. Furthermore, the National Board of Health counted the days until the patients gave consent to treatment, whereas we included the time until treatment was actually initiated. Further, we included the GPs’ suspicion of cancer and having noted so in the referral documents. The present study was hence able to demonstrate the importance of the referral process and the GPs’ knowledge, awareness and action. In addition, we were able to compare Vejle Hospital with other hospitals and identify aspects highlighting the possible impact of urgent referral on time to treatment.

In other healthcare settings, studies have reported a direct, negative impact on the length of the secondary care interval for non-urgent patients from the introduction of urgent referral for suspected cancer [19,27-29]. However, we saw no indication of such major adverse effect in our data.

## Conclusion

The secondary care interval decreased significantly after the national introduction of urgent referrals for suspected cancer. Patients with alarm symptoms in general and patients for whom the GP explicitly stated cancer suspicion saw the best effects of the urgent referral. Vejle Hospital remarkably managed to shorten their intervals even further. In conclusion, this study indicates that the shorter secondary care intervals result not only from the urgent referral policy, but also other factors are involved.

## Abbreviations

GP: General practitioner; IQI: InterQuartile interval; CI: Confidence interval.

## Competing interests

Co-author Frede Olesen is chairman of the Danish Cancer Society.

The other authors declare that they have no competing interests.

## Authors’ contributions

MBL participated in the design of the study, performed the statistical analyses and drafted the manuscript. RPH and DGH participated in the design of the study and helped to draft the manuscript. FO and PV participated in designing the study, assisted in data interpretation and made critical revision of the manuscript for important intellectual content. All authors read and approved the final manuscript.

## Pre-publication history

The pre-publication history for this paper can be accessed here:

http://www.biomedcentral.com/1472-6963/13/348/prepub
